# A system to analyze the initiation of random X-chromosome inactivation using time-lapse imaging of single cells

**DOI:** 10.1038/s41598-024-71105-y

**Published:** 2024-09-02

**Authors:** Manami Koshiguchi, Nao Yonezawa, Yu Hatano, Hikaru Suenaga, Kazuo Yamagata, Shin Kobayashi

**Affiliations:** 1 Cellular and Molecular Biotechnology Research Institute, National Institute of Advanced Industrial Science and Technology (AIST), 2-4-7 Aomi, Koutou-ku, Tokyo, 135-0064 Japan; 2 Faculty of Biology-Oriented Science and Technology, Kindai University, Kinokawa, Wakayama 649-6493 Japan; 3 Faculty of Life and Environmental Science, University of Yamanashi, Kofu, Yamanashi 400-8510 Japan; 4 Present Address: RIKEN Center for Biosystems Dynamics Research (BDR), Kobe, Hyogo 650-0047 Japan

**Keywords:** Epigenetics analysis, Embryogenesis, Dosage compensation

## Abstract

In female eutherian mammal development, X-chromosome inactivation (XCI) of one of the two X chromosomes is initiated early. Understanding the relationship between the initiation of XCI and cell fate is critical for understanding early female development and requires a system that can monitor XCI in single living cells. Traditional embryonic stem cells (ESCs) used for XCI studies often lose X chromosomes spontaneously during culture and differentiation, making accurate monitoring difficult. Additionally, most XCI assessment methods necessitate cell disruption, hindering cell fate tracking. We developed the Momiji (version 2) ESC line to address these difficulties, enabling real-time monitoring of X-chromosome activity via fluorescence. We inserted green and red fluorescent reporter genes and neomycin and puromycin resistance genes into the two X chromosomes of PGK12.1 ESCs, creating a female ESC line that retains two X chromosomes more faithfully during differentiation. Momiji (version 2) ESCs exhibit a more stable XX karyotype than other ESC lines, including the parental PGK12.1 line. This new tool offers valuable insights into the relationship between XCI and cell fate, improving our understanding of early female development.

## Introduction

In female eutherian or placental mammals, one of the two X chromosomes is randomly selected and inactivated immediately after implantation, an epigenetic process known as random X-chromosome inactivation (rXCI)^1,2^. Dysregulation of this regulatory mechanism results in the activation of both X chromosomes and leads to embryonic lethality, highlighting the importance of XCI in development^3–5^. Because only one of the two X chromosomes is chosen for inactivation in the rXCI process, it is widely believed that a mechanism exists by which female cells can count the number of X chromosomes, and all but one X chromosome is inactivated. Several models^6^ have been proposed to explain this “counting and choice” mechanism, but the explanations for this phenomenon remain controversial.

Considerable progress has been made in understanding the regulatory mechanisms involved in establishing rXCI^1,7–9^; however, the mechanism underlying the “counting and choice” of a single X chromosome has not been fully elucidated. The stochastic model is promising for the “counting and choice”^10^ mechanism. This model suggests that each X chromosome in the nucleus initiates XCI independently, as supported by the observation that, during the rXCI process, in addition to cells with one inactive X chromosome, some cells have both X chromosomes inactivated, whereas others have neither X chromosome inactivated. Cells displaying such noncompliant XCI patterns during the rXCI process can be expected to stop proliferating during differentiation because of the abnormal expression of genes on their X chromosome. Consequently, these cells will be lost from the proliferating cell population, and only cells with one appropriately inactivated X chromosome are likely to be selected for survival. This process could be regarded as a mechanism of “cell quality control during development,” whereby only cells adequately subjected to epigenetic regulation can survive.

One system can recapitulate in vitro the rXCI that occurs in the epiblast immediately after implantation by inducing the differentiation of embryonic stem cells (ESCs). Previous studies using this system have focused on analyzing gene expression in cell populations^11,12^ or single cells^13–15^ during differentiation. Because the epigenetic status of X chromosomes in each cell is thought to be differentially regulated when rXCI is established, analysis of cell populations is unsuitable for tracing individual cell fates in such heterogeneous populations. Moreover, single-cell RNA sequencing cannot follow the subsequent developmental fate of cells. When rXCI is established, cells with abnormal initiation of rXCI, i.e., cells with zero or two inactive X chromosomes (Xi), are expected to die during differentiation. However, these previous studies are so-called snapshots, and it is impracticable to follow living cell fate at the single-cell level via these methods.

We previously reported the Momiji mouse system^16,17^, which enables live-cell imaging of XCI using fluorescent protein expression as an indicator. This system employs eGFP and mCherry fluorescent reporters inserted into each X chromosome to detect epigenetic differences as differences in fluorescent color. By inducing ESC differentiation using the Momiji system, establishing rXCI over multiple cell divisions can be observed and analyzed via time-lapse imaging. However, the previously reported Momiji (version 1) ESC line has limitations: it cannot detect rXCI accurately because of the instability of the X chromosome, which is lost during differentiation in vitro. Here, we report a new Momiji (version 2) ESC line that maintains stable XX chromosomes during differentiation, and we developed a system that enables us to monitor the process of establishing the rXCI through time-lapse analysis. Using this system, we were able to verify that cell death occurs commonly during the differentiation process. Furthermore, it was suggested that the number of cells dying because of the failure of rXCI, if any, was small.

## Results

### B6 background Momiji (version 1) ESCs lose their X chromosome after repeated passage

**Fig. 1:**
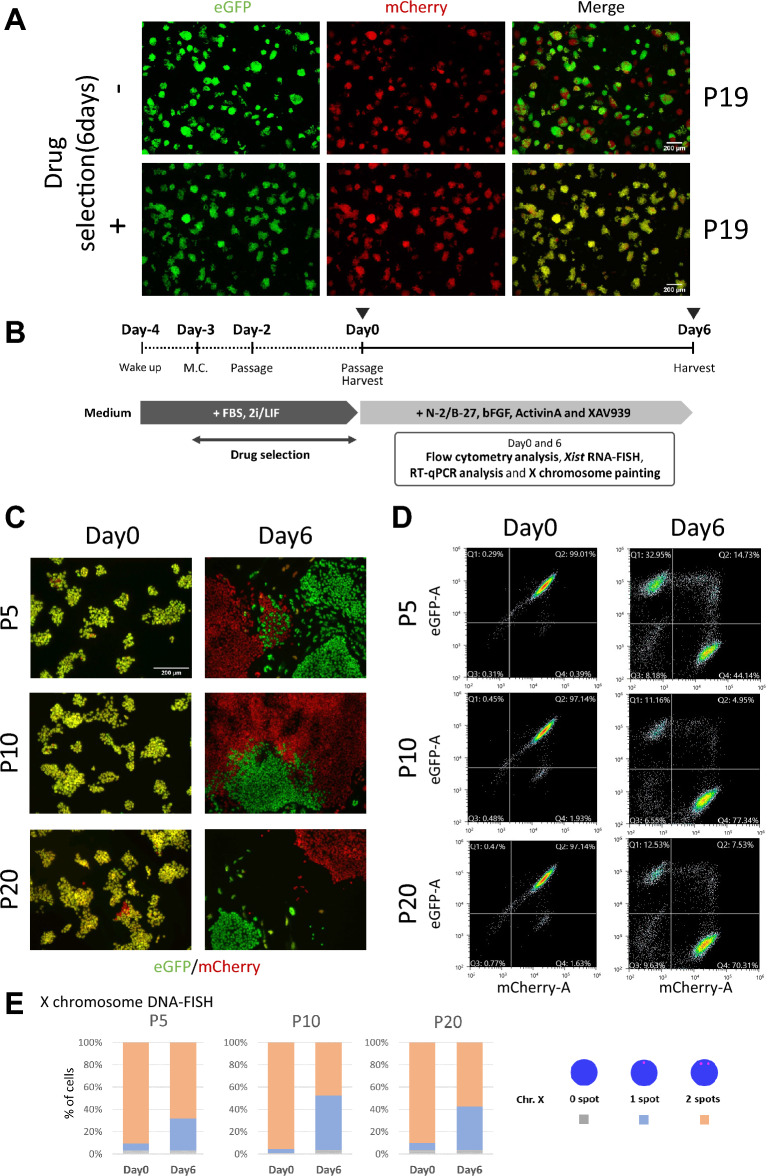
X-chromosome instability in Momiji (version 1) ESCs during culture for differentiation and maintenance. (**A**) Momiji (version 1) ESCs were maintained with repeated passages without drug selection (upper panels, total passage number 19) and after drug selection using puromycin and neomycin (lower panels, total passage number 19 including 6 days with drug selection). (**B**) A scheme for inducing Momiji (version 1) ESC differentiation. Drugs (neomycin and puromycin) were added only during ESC preculture and were removed from the medium after cell differentiation induction. This is because rXCI occurs after differentiation induction and one of the X chromosomes is inactivated, causing the cells to become drug resistant but only to puromycin or neomycin. MEK and GSK3 inhibitors are referred to as 2i. (**C**) Differentiation of Momiji (version 1) ESCs at passages 5, 10, and 20. (**D**) FACS analysis of Momiji (version 1) ESCs on day 6 of differentiation. (E) DNA-FISH analysis with an X-specific probe (No. MXO-10; Chromosome Science Labo) in Momiji (version 1) ESCs before and after differentiation. Three cell culture samples were hybridized independently. At least 100 nuclei were counted in each sample.

We previously established a 100% C57BL/6 N background ESC line named Momiji (version 1), whose X chromosomes were tagged with the fluorescent markers eGFP and mCherry to monitor the state of XCI in living cells^17^. First, we utilized the generated cell line to observe the process of establishing rXCI through time-lapse imaging. However, it is well known that the XX chromosome in ESC lines is unstable, and the X chromosome is frequently lost during maintenance and culture, resulting in a switch from the XX type to the XO type^18,21,22^. Before commencing our experiments, we investigated the stability of the X chromosome in the Momiji (version 1) ESCs. To assess the stability of the XX chromosome in these cells, we used the fluorescent markers eGFP and mCherry, which were inserted into each of the two X chromosomes (Fig. S1). Our observations indicated that the proportion of cells with XX-type chromosomes decreased with repeated passages (Fig. 1A, upper panels; the proportion of yellow cells was 47.4% (drug(–)), calculated from the number of pixels in the photo image), indicating that the ESCs had lost their X chromosomes. We also conducted drug selection for 6 days using two drug-resistance genes inserted into the two X chromosomes and confirmed that nearly 90% of the cells emitted yellow fluorescence after selection (Fig. 1A, lower panels; the proportion of yellow cells was 89.2% (drug( +), calculated from the number of pixels in the photo image). In the double drug (neomycin + puromycin) selection process, we selected cells with X^NeoR^X^PuroR^ chromosomes, and cells that had lost their X chromosome were excluded. We subsequently used these XX-type cells as starting materials for our differentiation experiments.

### Effect of the number of ESC passages on the loss of the X chromosome during differentiation

Next, we asked whether female XX ESCs lose their X chromosome during differentiation. We prepared Momiji (version 1) ESC lines at different passage numbers (passages 5, 10, and 20) and analyzed the effects of passage number on XX chromosome stability during differentiation. ESCs were differentiated into epiblast stem cells (EpiSCs) to recapitulate differentiation into epiblasts immediately after implantation to observe XCI initiation (Fig. 1B). Six days after inducing differentiation, the cell appearance changed to an EpiSC-like morphology, and the fluorescence shifted from yellow to monochromatic green or red (Fig. 1C,D). We then counted the number of X chromosomes in ESCs (day 0) and ESCs (day 6) using an X-specific DNA-FISH probe (Fig. 1E). Compared with those on day 0, on day 6, ESCs tended to lose their X chromosome during differentiation, and after differentiation ESCs (P10) and ESCs (P20) were more likely to lose their X chromosome than were ESCs (P5). These results suggest that for more accurate monitoring of the rXCI, it is better to avoid Momiji (version 1) ESCs at higher passages.

### Establishment of the Momiji (version 2) ESC model

**Fig. 2:**
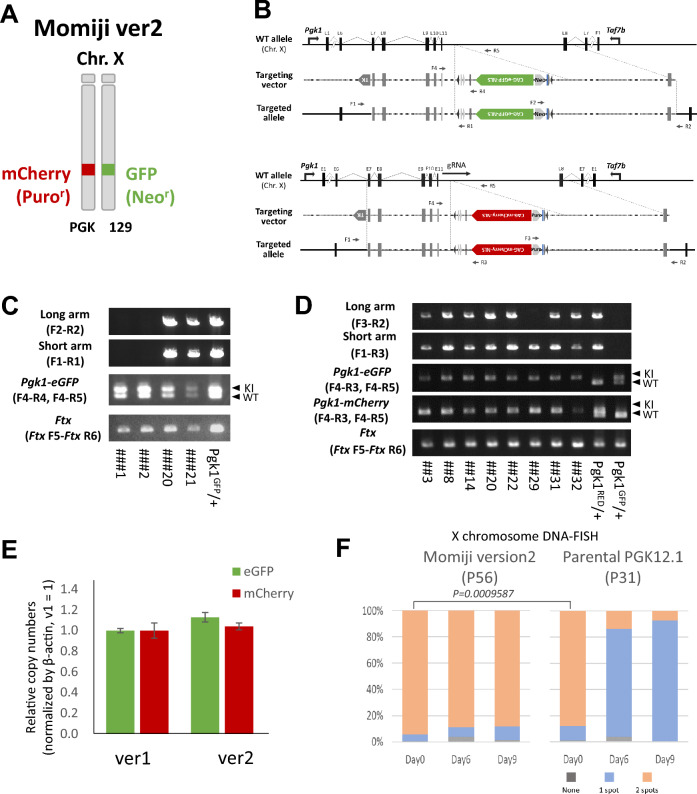
Targeting of fluorescence reporter genes in PGK12.1. (**A**) Schematic diagram of the Momiji (version 2) ESC genotype. (**B**) Strategy for targeting *eGFP* and *mCherry* reporters at the *Pgk1* locus. Genotyping of *eGFP* (**C**) and *mCherry* (**D**) in isolated cell lines (with grouping of gels cropped from different parts of the same gel or from different gels). Uncropped electrophoresis gel images are included in Figs. S6 and S7. (**E**) qPCR analysis of *eGFP* and *mCherry* in the genomic DNA of Momiji (versions 1 and 2). The genomic DNA obtained from Momiji (version 1) ESCs cultured under drug selection for 3 days was used as a control; this DNA had one copy insertion of the reporter gene in the X chromosome. (**F**) DNA-FISH analysis of Momiji (version 2) and parental PGK 12.1 ESCs using an X-specific probe (No. MXO-10, Chromosome Science Labo). Momiji (version 2) ESCs (P56) were cultured under undifferentiated conditions with drug selection (neomycin and puromycin) for 3 days and subsequently analyzed using DNA-FISH. The analysis used parental PGK12.1 ESCs (P31) without drug selection. Three cell culture samples were hybridized independently. At least 100 nuclei were counted in each sample. Differences between Momiji (version 2) and PGK12.1 on day 0 were determined using a Fisher exact test.

To overcome the problem of XX chromosome instability, we established a newly developed Momiji ESC cell line named Momiji (version 2) from the PGK12.1 ESC cell line (Fig. 2A). PGK12.1 ESCs are derived from a female embryo obtained by crossing 129 × (PGK × 129) F1 mice and are widely used in rXCI research^19^. To target PGK12.1 ESCs, CRISPR/Cas9 was used to cleave the chromosome near the homologous recombination site to improve the efficiency of homologous recombination (Fig. 2B). A CAG-*eGFP*-NLS reporter cassette with a Neo^R^ or a CAG-*mCherry*-NLS reporter cassette with Puro^R^ was inserted downstream of the *Pgk1* locus on each of the two X chromosomes. The results of genotyping of the homologous recombination of *eGFP* and *mCherry* reporters are shown in Fig. 2C,D. Quantification by qPCR predicted that both the *eGFP* and *mCherry* reporter genes had one copy of the reporter inserted (Fig. 2E). Next, we induced differentiation in modified Momiji (version 2) ESCs after removing XO cells by antibiotic selection. Using DNA-FISH analysis with an X-specific probe, we analyzed both Momiji (version 2) and parental PGK12.1 ESCs at days 0, 6, and 9 after inducing differentiation (Fig. 2F). On day 0, the proportion of XX ESCs was significantly greater in Momiji (version 2) ESCs than in PGK12.1, confirming that XO ESCs were effectively removed by the antibiotic selection in Momiji (version 2). The results from days 6 and 9 indicate that the stability of the X chromosome is much greater in Momiji (version 2) ESCs than in PGK12.1 ESCs after differentiation. The difference in starting proportions of XO cells before differentiation may lead to differences in X chromosome stability between the PGK12.1 and Momiji (version 2) cells during differentiation.

### X chromosomes in Momiji (version 2) ESCs are stable during the period of differentiation

**Fig. 3:**
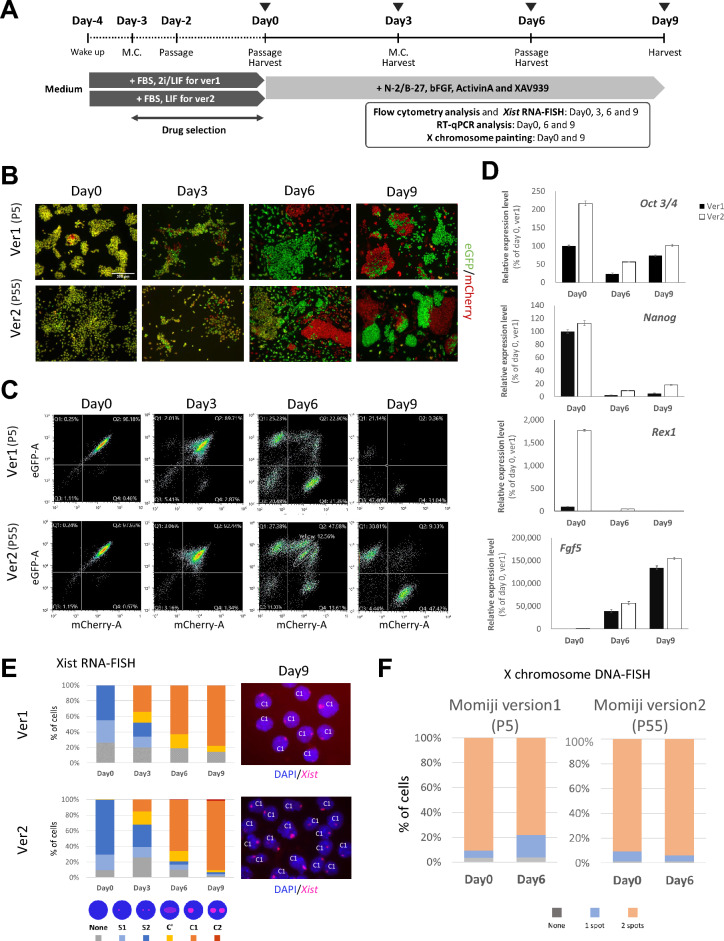
Improved X-chromosome stability of Momiji (version 2) ESCs during ESC differentiation. (**A**) A scheme for inducing differentiation in Momiji ESCs (versions 1 and 2). Momiji (version 1) ESCs were established under FBS + 2i/LIF conditions for efficiency. The cells (P5) were then used for experiments in which differentiation was induced without large expansions to avoid an increase in the passage number. By contrast, the parental strain PGK12.1 of Momiji (version 2) ESCs was established under conventional culture conditions, FBS-LIF, as described in the original report^19^. The same conditions were used for the Momiji (version 2) ESC culture experiments. Momiji (version 2) ESCs (P55) were used in this study. Temporal fluorescence analysis of eGFP and mCherry in Momiji (versions 1 and 2) ESCs over the period of differentiation by (**B**) fluorescence microscopy and (**C**) flow cytometry. (**D**) Temporal analysis of differentiation marker genes in Momiji (versions 1 and 2) ESCs on days 0, 6, and 9 of differentiation by RT‒qPCR (n = 3 or 4). (**E**) We conducted temporal analysis of *Xist* cloud formation in Momiji (versions 1 and 2) ESCs throughout differentiation using RNA-FISH. At least 40 nuclei were counted in each sample. Designations of the *Xist* pattern in each nucleus: None, no detectable *Xist* signal; S1, single spot signal; S2, two spot signals; C1′, large and faint single cloud; C1, single cloud; C2, two clouds. (**F**) DNA-FISH analysis of the X-chromosome number in Momiji (versions 1 and 2) ESCs using an X-specific probe (No. MXO-10, Chromosome Science Labo) before and after the period of differentiation. Three cell culture samples were hybridized independently. At least 100 nuclei were counted in each sample.

Next, we investigated whether rXCI could be monitored by inducing differentiation using Momiji (version 2) ESCs. Momiji (version 1) ESCs (P5) were used in parallel to facilitate comparisons (Fig. 3A). Upon inducing differentiation with XX cells, EpiSC-like colonies with a flat morphology were observed 9 days after initiating differentiation (Fig. 3B). FACS analysis revealed a gradual shift in fluorescence from yellow to monochromatic red and green between days 3 and 9 (Fig. 3C). While many cells displayed monochromatic red or green fluorescence 6 days after initiating differentiation, nonfluorescent and completely unchanged yellow fluorescent cells were also detected, suggesting the presence of cells with abnormal numbers of inactive X chromosomes (Fig. 3C, Momiji (version 2) day 6, lower panel; the proportions of nonfluorescent cells and yellow fluorescent cells indicated by the oval were 11.03% and 12.56%, respectively). As expected, the expression of the pluripotent stem cell markers *Oct3/4*, *Nanog*, and *Rex1* decreased during differentiation, whereas the expression of the ectoderm marker *Fgf5* increased, and the number of cells with an *Xist* cloud increased, confirming the differentiation of these cells into EpiSCs (Fig. 3D,E). Next, we checked which reporter (*eGFP* or *mCherry*) was inserted into the X chromosome. The red and green cells were collected on day 6, and the polymorphism of *Xist* expressed in each cell was examined. We confirmed that the *eGFP* reporter was inserted into the maternal 129 allele, and the *mCherry* reporter was inserted into the paternal PGK allele (Fig. S2). Finally, we analyzed the differentiated cells using DNA-FISH with an X-specific probe to assess X-chromosome loss during differentiation (Fig. 3F). Momiji (version 2) ESCs tended not to lose X chromosomes as often as version 1 ESCs (proportion of XO cells, version 1, 18.1%; version 2, 5.0%, count data *p* < 0.001 using a Fisher exact test), indicating better X-chromosome stability in Momiji (version 2). These results and those of Fig. 2F indicate that Momiji (version 2) ESCs offer greater advantages than other ESC lines for monitoring rXCI because they retain two X chromosomes more stably during differentiation.

### Random XCI lineage tree from a single-cell Momiji (version 2) ESC

**Fig. 4:**
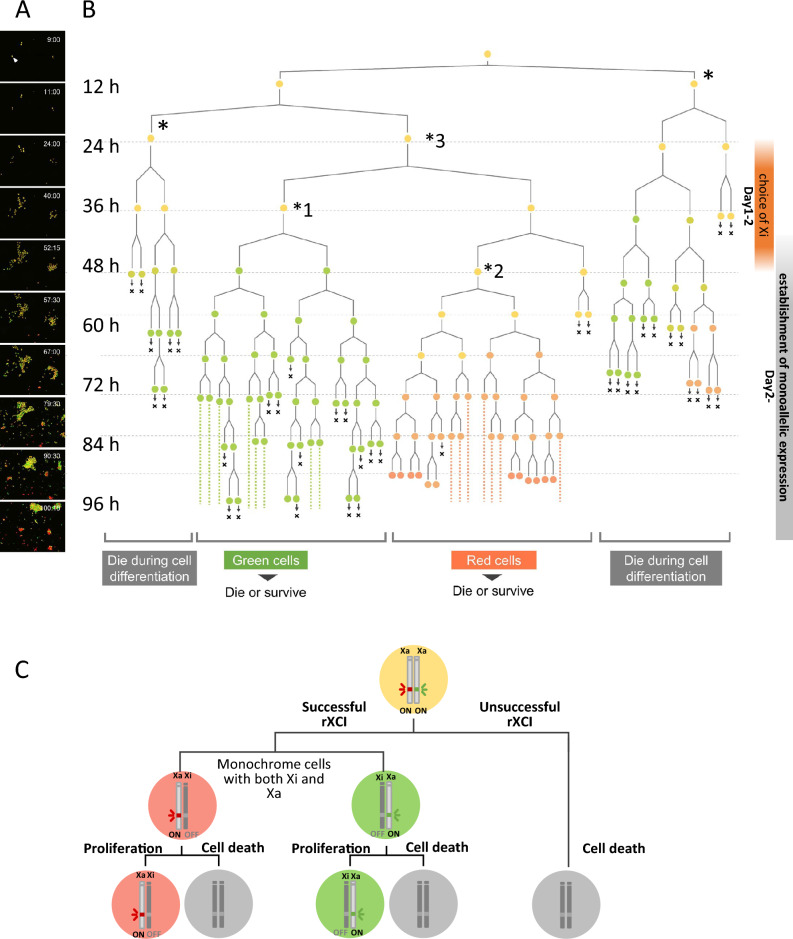
Random XCI lineage tree from a single cell of Momiji (version 2) ESCs. (**A**) Still images captured from video sequences of live-cell imaging during differentiation of Momiji (version 2) ESCs. (**B**) Lineage tree of Momiji (version 2) ESCs during differentiation. The cell division was tracked, and the cell color was determined manually. All progeny cells, which were derived from a single cell marked with *, died during cell differentiation. All differentiated cells derived from one yellow cell (marked with *1) turned green, indicating that Xi choice was already determined in the *1 cell. Similarly, Xi choice was determined for yellow cells (*2), from which all the derived cells turned red. Xi choice was not determined in yellow cells (*3), which are progenitors of yellow cells *1 and *2. These patterns predict that the Xi choice is determined during cell proliferation between cells *1/*2 and *3 (i.e., approximately the first and second days after inducing differentiation). (**C**) Summary of the relationship between the rXCI process and cell fate in a single cell.

We induced differentiation of Momiji (version 2) ESCs into EpiSCs and performed time-lapse imaging for 4 days (Fig. 4A, Supplementary Movie S1). We analyzed three independent ESCs and constructed cell lineage trees derived from one ESC, as shown in Fig. 4B (redrawn figure for cell no. 1) and Supplementary Fig. S3A–C for cell nos. 1, 2, and 3, respectively. Although a considerable proportion of cells died during differentiation (Fig. S4; the number of dead cells was counted at 72, 74, 76, and 78 h), such cells often arose from a single cell derived from a branch of the lineage tree (Fig. 4B, marked with *). By observing the cells as their fluorescence shifted to green or red, we determined the time course for the inactivation of each X chromosome. In this analysis, the timing of rXCI establishment was determined based on the silencing of the reporter. We found that rXCI was established approximately 2 days after inducing differentiation, although there was considerable variation among the individual cells. From the branching pattern of the lineage tree, we inferred that “Xi choice,” which determines which X chromosome will be inactivated, occurred approximately between the first and second days after inducing differentiation (details are found in the legend to Fig. 4B). At this point, the cell emitted yellow fluorescence, but its fate, i.e., whether its fluorescence would turn red or green, had already been determined. Whether Xi choice precedes *Xist* upregulation or *Xist* upregulation precedes Xi choice in the initiation step of XCI is controversial^6,20^. We examined this controversial kinetics using our Momiji (version 2) ESC system. *Xist* RNA-FISH results showed that *Xist* upregulation occurred from days 0 to 3 after ESC differentiation (Fig. 3E) and that the timing of *Xist* upregulation and Xi choice (approximately from days 1 to 2 after differentiation induction as predicted by our live imaging) were almost identical. Which of these events precedes the other remains unclear and requires a more detailed elucidation of the molecular mechanisms involved.

Notably, after 2 days of differentiation, several yellow fluorescent cells died while rXCI was being established (Fig. S3A–C, marked with *), and these cells tended to exist in clusters within a tree. The summary diagram in Fig. 4C shows the relationship between rXCI establishment and cell death. Through this time-lapse analysis, we demonstrated that a considerable number of cells undergo cell death during differentiation. Cell death was observed both in cells that initiated rXCI normally and those that did not.

### Comparison of the cell death of XX and XO ESCs induced during differentiation

Based on the assumption that XCI during differentiation is crucial, it is generally expected that cells failing to establish rXCI will undergo cell death during differentiation. To determine how many cells die due to such rXCI defects, we induced the differentiation of XX ESCs (Momiji (version 2)) and XO ESCs (PGK12.1_XO) and measured cell death over 6 days using DAPI staining. XO ESCs are subclones of PGK12.1 ESCs and are cells that have lost one X chromosome, becoming XO (Fig. S5A). If there is a difference in the rate of cell death between XX and XO cells, it can be inferred that cell death is due to rXCI failure. In both XX and XO cells we found that cell death peaked at days 3–4 after differentiation was induced, but cell death occurred slightly earlier in the XO cells (Fig. S5B,C). There was no obvious difference in the proportion of cell death between the two cell lines, suggesting that the failure to establish rXCI does not account for a large percentage of cell deaths.

## Discussion

Many existing methods used to analyze rXCI status, including gene expression analysis, immunostaining, and RNA-FISH analysis, involve cell destruction, making it difficult to follow the progress of rXCI over time and clarify its relationship to cell fate. With the development of Momiji (version 2) ESCs, it is now possible to confirm cell fate while tracking rXCI in a single cell. Using this cell line, we overcame the problem of XX chromosomal instability during ESC differentiation by targeting PGK12.1 ESCs. When we monitor the expression of fluorescent proteins, one of the issues in live imaging is their half-life. Protein accumulation can cause a gap in the actual timing of transcription, making accurate measurements challenging. In the present study, ESCs were induced to differentiate into EpiSCs, and rXCI was monitored. Both cell types used in this study were stem cells with a high division rate: ESCs (one division: 10 h (40 frames) in this observation) and EpiSCs (one division: 7.5 h (30 frames)). Therefore, fluorescent proteins that accumulate in the nucleus are released from the nucleus at each cell division, which provides the advantage that gene expression can be detected with only a slight time lag.

The Momiji (version 2) ESC line has an additional feature that enables allelic expression analysis of X chromosomes using DNA polymorphisms. By tracking a fluorescent reporter inserted into the *Pgk1* locus, this ESC system can monitor the status of a single locus. Furthermore, polymorphisms on X chromosomes can be used to distinguish the expression of paternal X-linked genes from maternal genes. In Momiji (version 2) ESCs, the paternal X chromosome, derived from the PGK strain, is marked with mCherry, whereas the maternal X chromosome, derived from the 129 strain, is marked with eGFP. Thus, DNA polymorphisms between the two strains, when combined with cellular fluorescence, can be used to distinguish the expression of several hundred X-linked genes.

X-chromosome loss, or “X instability,” is commonly observed over successive passage of mouse ESCs^18,21,22^. In our experiments, Momiji (version 1) ESCs, which were established using inbred B6N background embryos with 2 inhibitors (MEK and GSK3 inhibitors) in the medium, tended to lose the X chromosome more than Momiji (version 2) ESCs, which were established using F1 hybrid background embryos without the inhibitors in the medium. We cannot exclude the possibility that X-chromosome stability, especially from a B6N inbred background, may vary from clone to clone and from batch to batch of ESCs. Additionally, ESC derivation and culture conditions, such as the presence or absence of the two inhibitors, may also affect stability. However, empirical observations have shown that many inbred mouse ESCs tend to lose their X chromosome, whereas ESCs established from F1 hybrid crosses tend to retain their XX chromosome stably^23^. Nevertheless, why F1 hybrid ESCs maintain the XX chromosome more stably than inbred ESCs remains unclear. A potential explanation includes genome-wide hypomethylation, which is distinctively observed in female ESCs but not in male ESCs^24–26^. Elucidating the mechanisms that maintain X stability, including DNA methylation as a possible cause, is warranted.

When we examined the induction of differentiation into EpiSCs, we observed a significant loss of X chromosomes even in PGK12.1 under our experimental conditions. By contrast, Momiji (version 2) ESCs maintained greater stability of the X chromosome (approximately 90%). This finding suggested that factors other than the F1 hybrid genetic background may be responsible for the X-chromosome stability during differentiation. One possibility is the presence of drug-resistance cassettes inserted into the X chromosomes. Unlike the parental PGK12.1 line, XO cells were removed from the Momiji (version 2) line by drug selection during preculture. It is possible that a slight contamination of XO cells before inducing differentiation may have decreased the proportion of XX cells during differentiation. However, in the Momiji (version 1) line, where the same drug selection was used, only 50%–80% of cells maintained the two X chromosomes during differentiation. These results suggest that in Momiji (version 2) ESCs, combining the F1 hybrid genetic background and drug selection results in greater stability and maintenance of the XX chromosome after inducing differentiation.

Many cells die during ESC differentiation, possibly through apoptosis^27–29^. Our live-cell observations revealed that cells derived from the same single cell suffer the same fate and die. Cell death can occur in all cells derived from a single cell if (i) chromosomal aberrations, including aneuploidy, occur; (ii) epigenetic dysregulation occurs in the autosome; or (iii) rXCI fails to be established during cell differentiation. Our lineage tree observations suggest that once these abnormalities occur, they affect the fate of daughter cells and all their descendants, and they appear to be irreversible, with abnormal cells dying during differentiation.

Additionally, distinguishing between the various causes of cell death during differentiation is difficult. Therefore, the mechanism of cell death resulting from abnormal rXCI during the rXCI process remains poorly investigated. In the present study, we compared cell death between XX and XO ESCs and found that cells dying spontaneously while establishing rXCI do not account for a large proportion of overall cell death. These findings suggest that the failure to initiate rXCI is not a primary cause of the cell death that occurred during differentiation. Notably, on day 6 of differentiation, FACS analysis revealed that approximately 12% of the cells remained entirely fluorescent yellow, and another 11% remained nonfluorescent (Fig. 3C version 2, day 6), as predicted by the stochastic model of rXCI. These findings suggested that establishing rXCI may involve stochastic *Xist* expression resulting in 0 (yellow fluorescent cells), 1 (monochromatic red or green fluorescent cells), or 2 (nonfluorescent cells) inactive X chromosomes. The Momiji (version 2) ESC line enables the separation of cells with accurate rXCI and aberrant rXCI patterns while keeping the cells viable during the rXCI process. Determining the relationship between these cells and *Xist* expression and whether abnormal rXCI cells with 0 or 2 *Xist* expression undergo apoptosis or necrosis upon differentiation is highly informative. Understanding the mechanisms underlying the exclusion of such cells with defective rXCI is critical for understanding female mammalian development. Our newly developed Momiji (version 2) ESC platform allows for a more detailed analysis of these phenomena.

Although ESC differentiation in vitro is widely used to mimic fetal development, the extent to which the system in vitro reflects rXCI in vivo is unknown (including the cell death that occurs when establishing rXCI and the timing of Xi choice). Although there are reports of single-cell sequencing analysis of rXCI initiation in vivo^30^, additional direct observation of the rXCI establishment process via live imaging in vivo could elucidate what occurs when establishing rXCI in vivo. However, this remains a challenge.

## Methods

### Derivation of Momiji (version 1) ESCs and culture conditions

Momiji (version 1) ESC lines were established from E3.5 blastocysts derived from mating *Pgk1*^*RED*^/+ female (100% C57BL/6 N background) and *Pgk1*^*GFP*^/Y male (100% C57BL/6 N background) mice, as described previously^17^. In brief, the expanded blastocysts were plated into gelatin-coated dishes with monolayers of mouse embryonic fibroblasts (MEFs). To derive ESCs, we used ESC medium consisting of KnockOut DMEM (Thermo Fisher Scientific) supplemented with 20% fetal bovine serum (Thermo Fisher Scientific), nonessential amino acids (0.1 mM) (Thermo Fisher Scientific), 1 × nucleosides (Merck), 2-mercaptoethanol (0.1 mM) (Thermo Fisher Scientific), penicillin–streptomycin–glutamine solution (0.5 mg/mL) (Thermo Fisher Scientific), mouse LIF (3000 U/mL; catalog no. ESG1107; Merck), and two inhibitors (1 μM PD0325901 for GSK-3 (Fujifilm Wako) and 3 μM CHIR99021 for MEK (Axon MedChem)). The inner cell mass outgrowths were dissociated 10 days after plating and replated on MEFs. Undifferentiated ESC colonies were expanded to establish ESC lines, and feeder-free ESCs were used for further analysis. Both the Momiji (versions 1 and 2) ESC lines were cultured in ESC medium as described above at 37 °C in humidified air with 5% CO_2_. To culture PGK12.1 ESCs and Momiji (version 2) ESCs, two inhibitors (1 μM PD0325901 and 3 μM CHIR99021) were excluded from the medium. Mice were euthanized by excessive inhalation of high-concentration isoflurane and confirmed dead by cervical dislocation.

### Targeting vector and gRNA expression vector for establishing Momiji (version 2)

Targeting vectors were constructed as previously reported^17^. In brief, a cassette of *eGFP* or *mCherry* reporter genes was inserted into the downstream sequence of *Pgk1* exon 11 (Fig. 2A or B). The neomycin and puromycin drug-resistance gene markers were also inserted into the targeted locus with *eGFP* and *mCherry* reporter cassettes, respectively. The gRNA sequence, which recognizes the downstream sequence of the *Pgk1* locus, was cloned and inserted into pX330. The gRNA oligonucleotides used are listed in Supplementary Table S1.

### Targeting of reporter genes to the X chromosome of PGK12.1

PGK12.1 ESCs were kindly provided by Brockdorff^19^. gRNA/Cas9-mediated double-strand breaks and subsequent homologous recombination with a targeting vector were used to target reporter cassettes into the *Pgk1* locus of PGK12.1 ESCs. We introduced mutations into the PAM sequences of the targeting vector to prevent cleavage after homologous recombination. PGK12.1 cells were seeded on a 6-well plate and transfected with the targeting vector or gRNA expression vector using Lipofectamine LTX with Plus Reagent (Thermo Fisher Scientific). Before transfection, the targeting vectors were digested with NotI (New England Biolabs) for linearization. We started drug selection with neomycin (250 μg/mL) (G418; Sigma Aldrich) for *eGFP* or puromycin (0.4 μg/mL) (Invitrogen) for *mCherry* 18 h after transfection. We added ganciclovir (3 μM) (Denosine; Mitsubishi Tanabe Pharma) to the medium 24 h after starting drug selection. After 5 days of drug selection, the cells were plated on feeder cells in 6-well plates to isolate single colonies. After 5 days of incubation, each colony was picked and transferred to a well in a 96-well plate coated with feeder cells. Genomic DNA was extracted from isolated cells using a Wizard SV genome DNA purification system (Promega) and was genotyped using specific PCR primers, as listed in Table S1.

### Cell differentiation

Momiji (versions 1 and 2) ESCs were cultured in EpiSC medium (DMEM/F-12 (1:1) supplemented with HEPES and N-2, B-27, MEM supplemented with nonessential amino acids, GlutaMax, 2-mercaptoethanol (Thermo Fisher Scientific), basic fibronectin growth factor (bFGF) (20 ng/mL) (Fujifilm Wako), activin A (20 ng/mL) (R&D Systems), and XAV939 (10 μM) (Sigma Aldrich)) for 6–9 days. The expression of fluorescent proteins was observed using a fluorescence microscope. The cells were passaged on day 6, after which observation continued until day 9.

### X-chromosome-specific DNA-FISH analysis

The cells were harvested at 0, 3, 6, and 9 days after the beginning of differentiation and were fixed with Carnoy solution (acetate diluted 1:3 (v/v) with ethanol). The fixed cells were spotted on glass slides and hybridized with a DNA probe specific for mouse chromosome X (No. MXO-10, Chromosome Science Labo) overnight at 37 °C. After hybridization, the slides were washed with 50% formamide/2 × SSC three times and 1 × SSC once and mounted with ProLong Diamond Antifade Mountant with DAPI (Thermo Fisher Scientific). The hybridized probes were observed using a fluorescence microscope.

### RNA-FISH

Cells were fixed using the same method as that used for X-chromosome-specific FISH analysis and spotted on glass slides. The cells on the slides were pretreated with 0.5% Triton X-100/PBS(–) and then dehydrated with 70% ethanol, followed by 100% ethanol. Dehydrated cells were incubated with an *Xist*-specific probe prepared by nick translation with Cy3-dCTP (GE Healthcare) from pXist 1986–9498 at 42 °C overnight. After hybridization, the slides were washed with 50% formamide/2 × SSC three times and with 1 × SSC three times. The hybridization was observed using a fluorescence microscope.

### Flow cytometry analysis

Harvested cells were fixed with 4% formaldehyde for 15 min at room temperature. The fixed cells were suspended in PB1 medium (Kyudo, Saga, Japan) and analyzed by flow cytometry on an SH800 instrument (Sony).

### Real-time PCR

Total RNA was extracted from cells using an RNeasy Micro Kit (Qiagen) and reverse transcribed with SuperScript IV VILO Master Mix with ezDNase (Thermo Fisher Scientific). The synthesized cDNA was amplified and detected using StepOne Plus (Thermo Fisher Scientific). The sequences of the primers used for real-time PCR are listed in Table S1.

### Live-cell imaging

Momiji ESCs were seeded on 35-mm glass-bottomed dishes (Mat Tek Corp. P35G-1.5-14-C) at 0.4 × 10^5^ cells/dish in 4 mL of EpiSCM. The cells were imaged three-dimensionally (3D) using a boxed-type confocal laser microscope with an incubation chamber (CV1000, Yokogawa Electric Corp.) set at 37.0 °C in 5% CO_2_, 20% O_2_, and 75% N_2_ with saturated humidity. The medium was changed every day; 3 to 4 mL of medium was discarded, and 3 mL of new EpiSCM was added. Images were taken at 15-min intervals for 7 days and 34 μm along the Z axis (1-μm intervals × 35 slices) using the following laser parameters: excitation, 488 nm and 561 nm; emission, 525/50 nm and 617/73 nm; and power emitted from the objective, 0.05 mW and 0.10 mW, respectively. We used a 20 × oil-immersion objective (UPLSAPO20X: NA 0.85; Olympus). The exposure time was 100 ms, and the camera gain was set at 75–80%. The images were acquired with 4 × 5 tiles. Each frame was checked manually to track cell division, and an observer determined the color of the cells visually to construct the cell lineage tree from a single cell.

### Allelic expression analysis

Differentiation of Momiji (version 2) ESCs was induced as described above. After 6 days of treatment, the cells were sorted according to their fluorescence using a FACS instrument. Total RNA extracted from yellow, green, and red fluorescent cells was reverse transcribed and amplified using specific primers for a fragment of *Xist*, including the locus with single nucleotide polymorphisms between the 129 and PGK alleles. The sequences of these amplicons were determined after purification via gel extraction.

### Cell death assay

The XO subclone PGK12.1_XO (E8) cell line, which has the same genetic background as PGK12.1, was kindly provided by Neil Brockdorff^19,25^. The samples were prepared according to previously described methods with modifications of DAPI staining^31^. Briefly, cells removed from the culture dish were added to the culture supernatant and centrifuged. After washing with PBS, the dead cells were stained with DAPI, and the number of stained cells was counted using a fluorescence microscope.

### Statistical analysis

All the values are presented as the means ± SEMs. Statistical analysis was performed using R. *P* < 0.05 was considered to indicate significance.

### Supplementary Information

Supplementary Video 1.

Supplementary Information 1.

## Data Availability

The datasets analyzed during the current study are available from the corresponding author upon reasonable request.
