# Supplementary material for: A system to analyze the initiation of random X-chromosome inactivation using time-lapse imaging of single cells
Source: Sci Rep. 2024 Sep 2;14:20327. doi: 10.1038/s41598-024-71105-y (PMC11369159; doi:10.1038/s41598-024-71105-y)
Supplement: Supplementary file 2 — Supplementary Information 1. [file 41598_2024_71105_MOESM2_ESM.pdf]

Supplementary information

A system to analyze the initiation of random X-chromosome inactivation using time-lapse imaging of single cells

Manami Koshiguchi, Nao Yonezawa, , Yu Hatano,
Hikaru Suenaga, Kazuo Yamagata, Shin Kobayashi

Supplementary information includes
Supplementary Figure S1- S7.
Supplementary Table S1- S2.

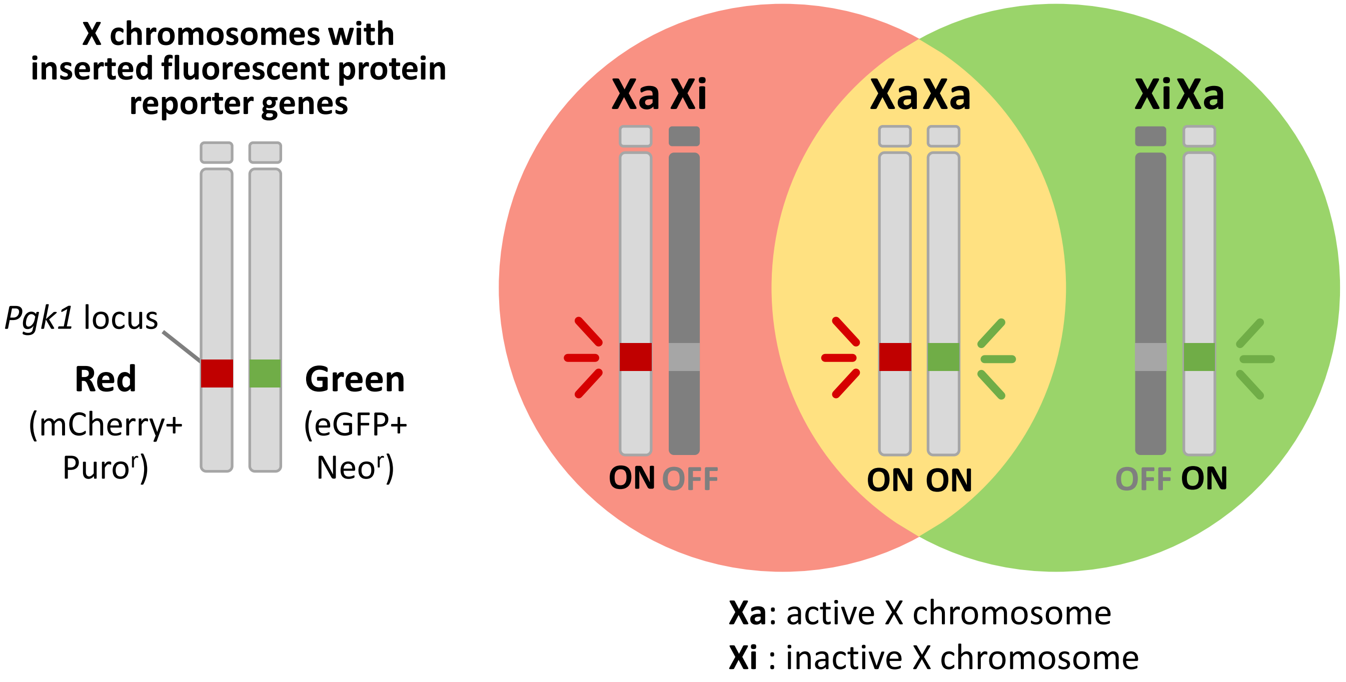

Figure S1. Overview of the Momiji system

In the Momiji system, *mCherry* and *eGFP*, which code for red and green fluorescent reporter proteins, respectively, were inserted into the locus for the *Pgk1* gene on each X chromosome. When either of the alleles was inactivated (XaXi or XiXa), the reporter protein was expressed monoallelically. In this case, the cells fluoresced red or green. When both X chromosomes are active (XaXa), the cells are detected as yellow due to biallelic expression of reporter proteins. This system enables visualization of X-chromosome activity by observation of fluorescent reporter proteins and facilitates temporal assessment of rXCI in living cells.

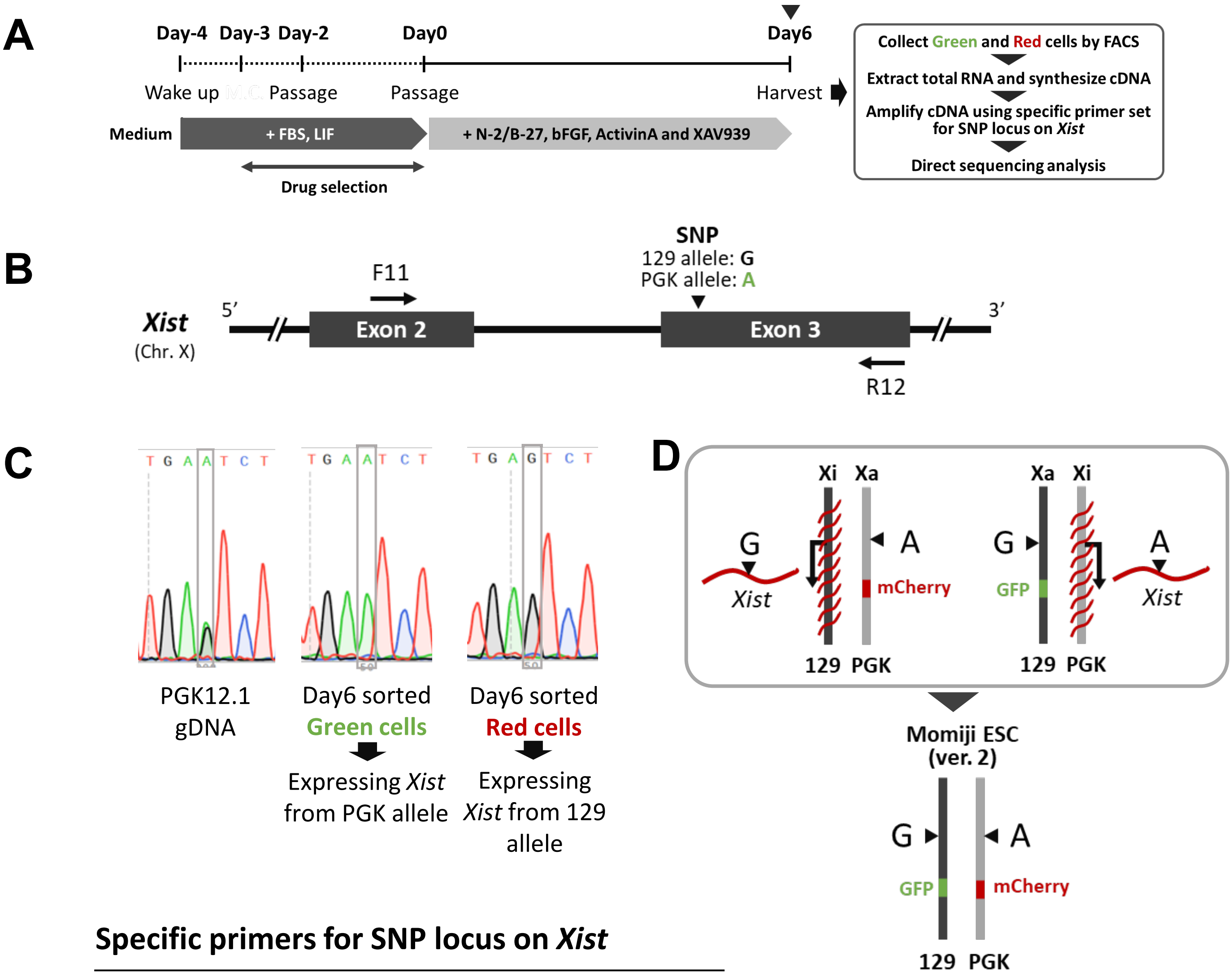

Specific primers for SNP locus on *Xist*

F11	5′- GCCTCAAGAAGAAGGATTGCCTGGA -3′
R12	5′- CCATGTGGCCTGTAAGCTTTTTGTTCA -3′

Figure S2. Allelic expression analysis of *Xist* in Momiji (version 2) ESC

(A) A scheme of allele determination of Momiji (version 2) ESC. After 6 days of differentiation induction, green and red fluorescent cells were collected, and total RNA extracted from the cells was reverse transcribed and amplified using specific primers for the SNP locus on *Xist*. The amplified fragments were sequenced to determine the allele in which the *eGFP* or *mCherry* reporter was inserted.
(B) Location of an SNP on the *Xist* gene. On the locus, a single G/A substitution, G on the 129 allele and A on the PGK allele, was used to distinguish each allele. A specific primer set (F11 and F12) was used for the amplification of fragments containing the polymorphism.
(C) Direct sequencing analysis of the SNP-containing fragments amplified from sorted monochromatic cells. In green and red cells, *Xist* was expressed from the PGK allele and 129 allele, respectively.
(D) An illustration of allelic determination of Momiji (version 2) ESCs.

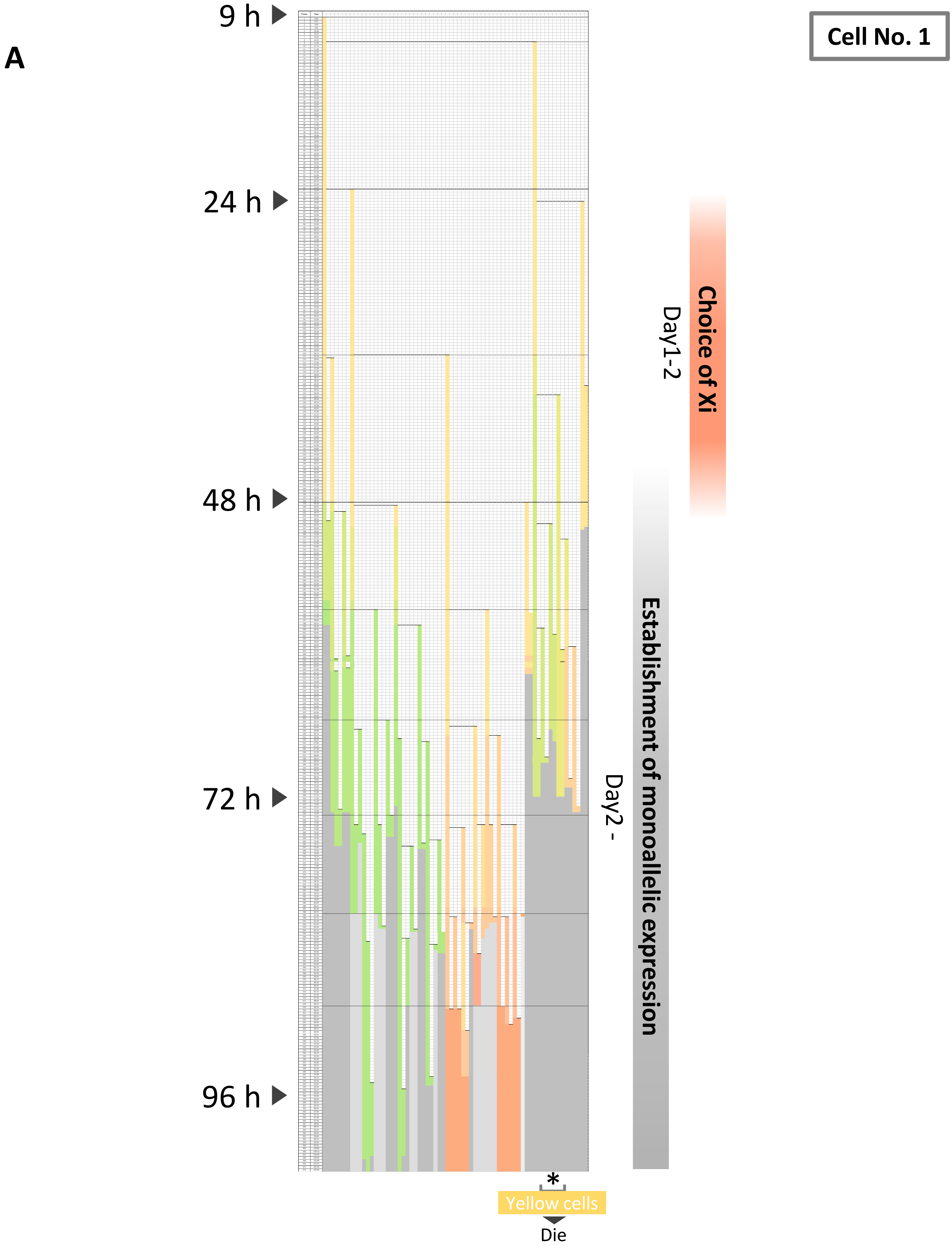

Figure S3. Random XCI lineage tree from a single cell of Momiji (version 2) ESC (cell Nos. 1–3) (A) Trees for cell No. 1, (B) for cell No. 2, and (C) for cell No. 3. In some branches, cells died while maintaining two active X chromosomes (indicated by *). Colors reflect the fluorescence of cells. Dark gray and dot patterns indicate “dead cells” and “untraceable cells,” respectively.

B

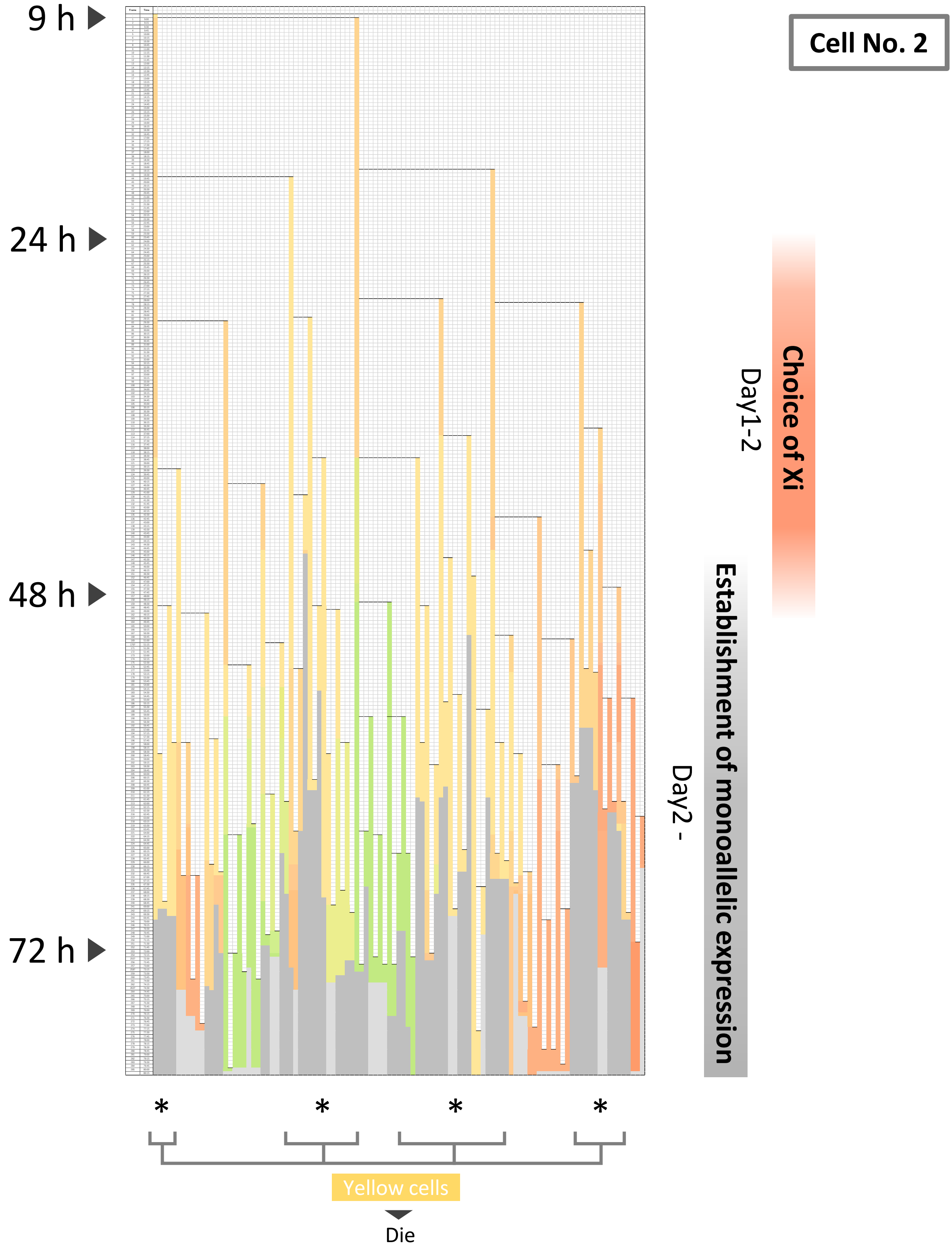

Figure S3. Random XCI lineage tree from a single cell of Momiji (version 2) ESC (cell Nos. 1–3)

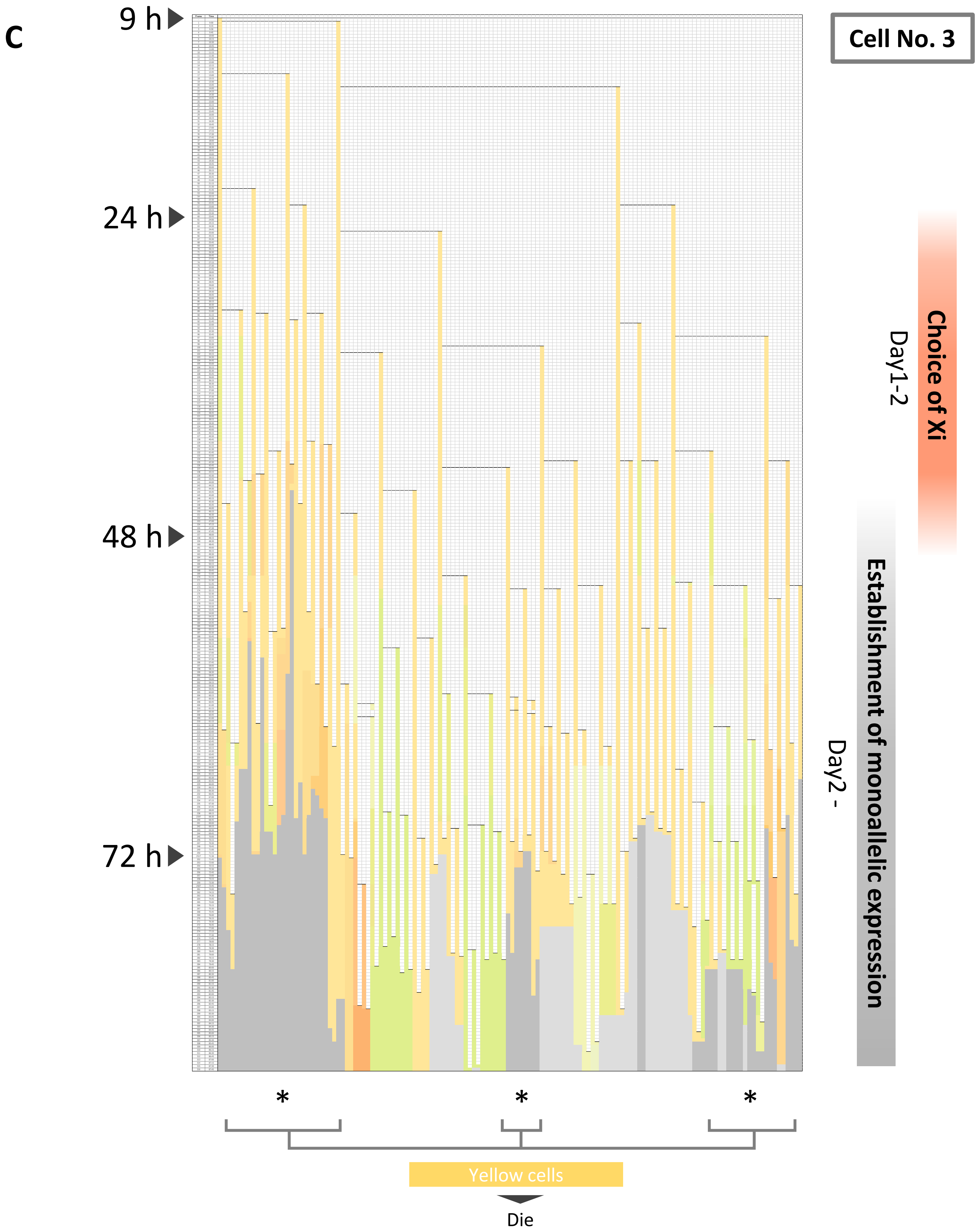

Figure S3. Random XCI lineage tree from a single cell of Momiji (version 2) ESC (cell Nos. 1–3)

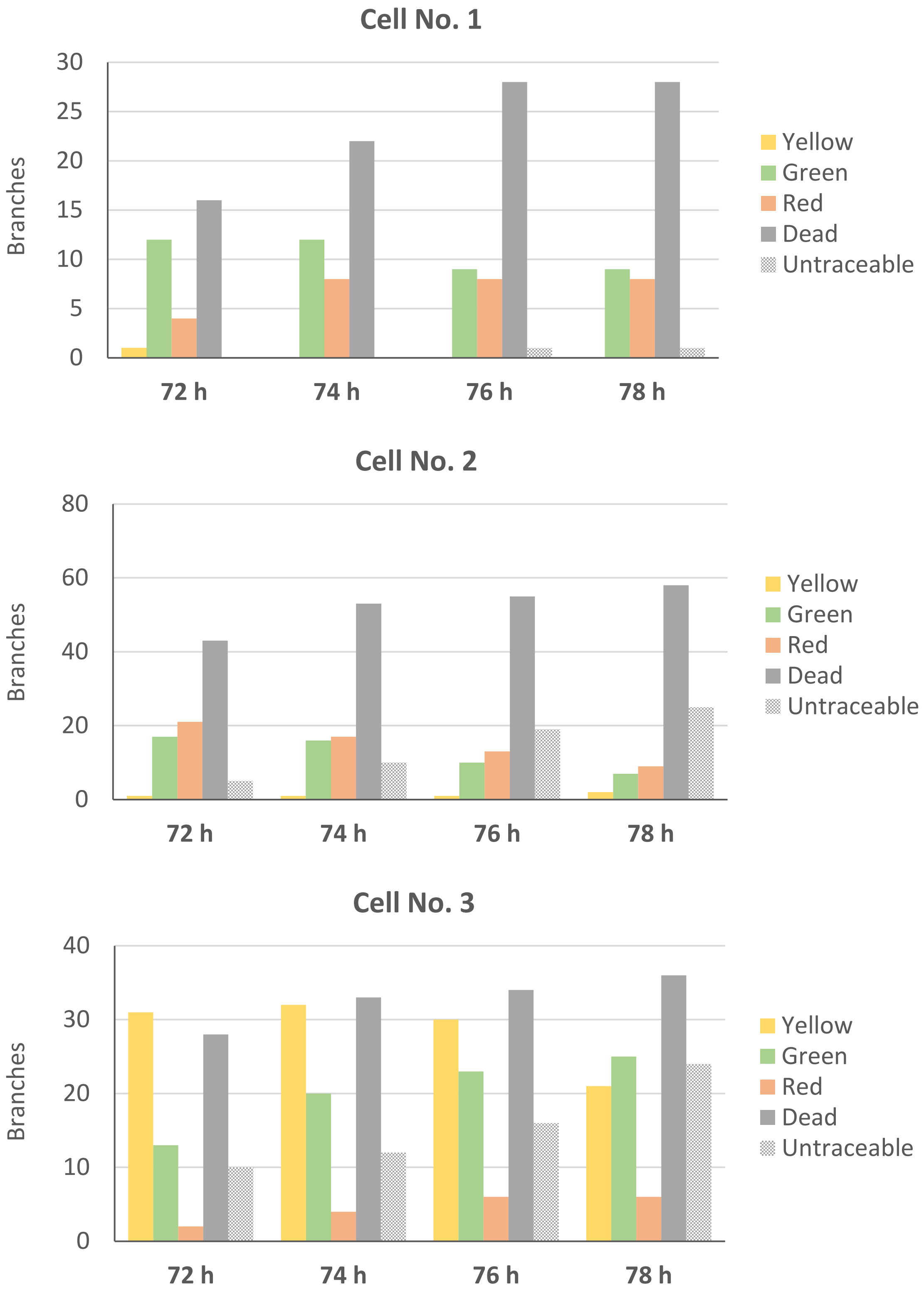

Figure S4. Cell counts of the lineage tree from a single cell of Momiji (version 2) ESC (cell Nos. 1–3)
The number of yellow, green, red, and dead cells at 72, 74, 76, and 78 h after differentiation induction are shown; cell counting was based on the measurements described in Figure S3.

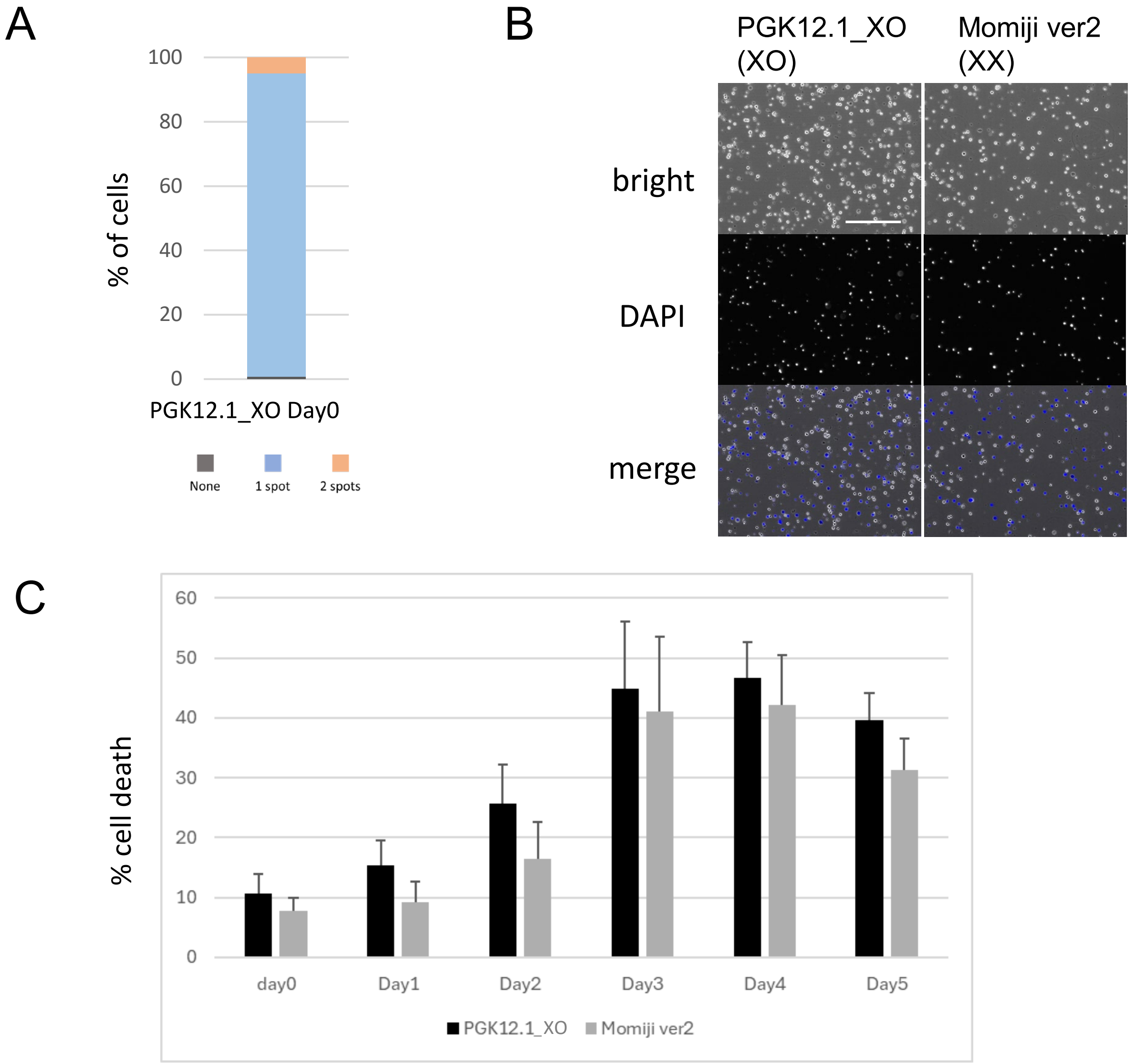

Figure S5, Assay of cell death in PGK12.1_XO and Momiji (version 2) ESCs during differentiation

(A) DNA-FISH analysis with an X-specific probe (catalog no. MXO-10; Chromosome Science Labo) in PGK12.1_XO ESCs before differentiation. One cell culture sample (n = 1) was hybridized, and at least 100 nuclei were counted. (B) Cell death on day 3 after initiating differentiation was visualized with DAPI staining. Scale bar, 50 μm. (C) DAPI-positive cells are shown as a percentage of the total cells per field. Three cell culture samples were measured independently, with at least 100 cells counted in each sample.

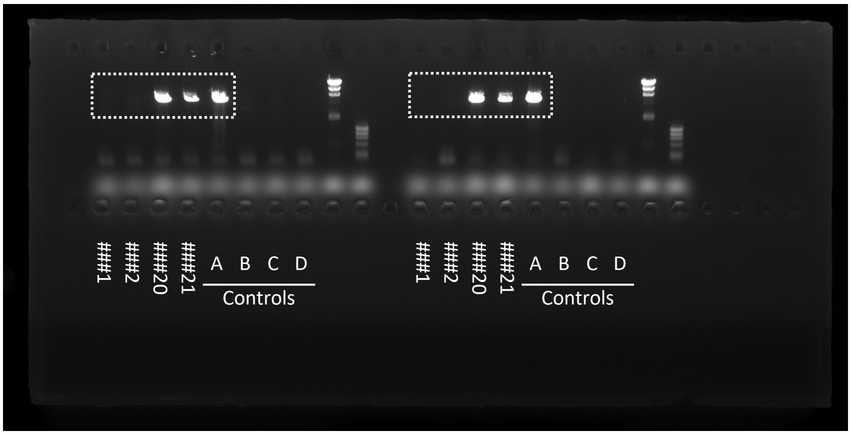

A: #99(PG/+)
B: PGK12.1-WT
C: PGK12.1-TK(+) #1
D: XGFP-TV

Long arm **Short arm**

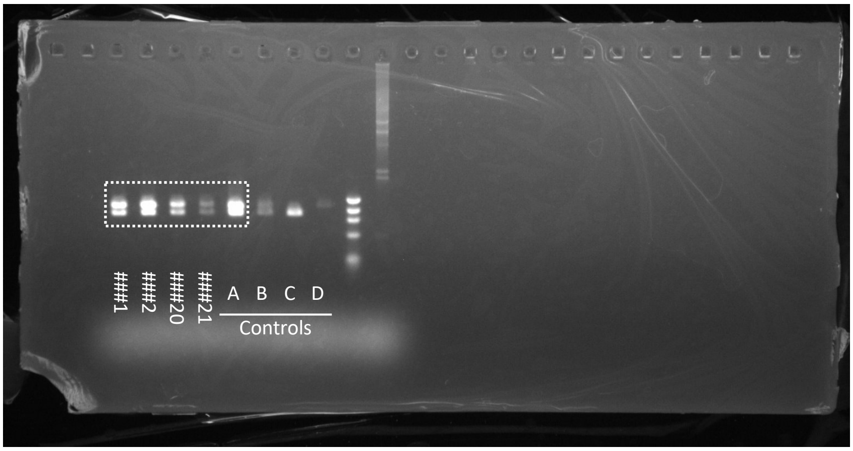

A: #99(PG/+)
B: PGK12.1-TK(+) #1
C: PGK12.1-WT
D: XGFP-TV

Pgk-eGFP

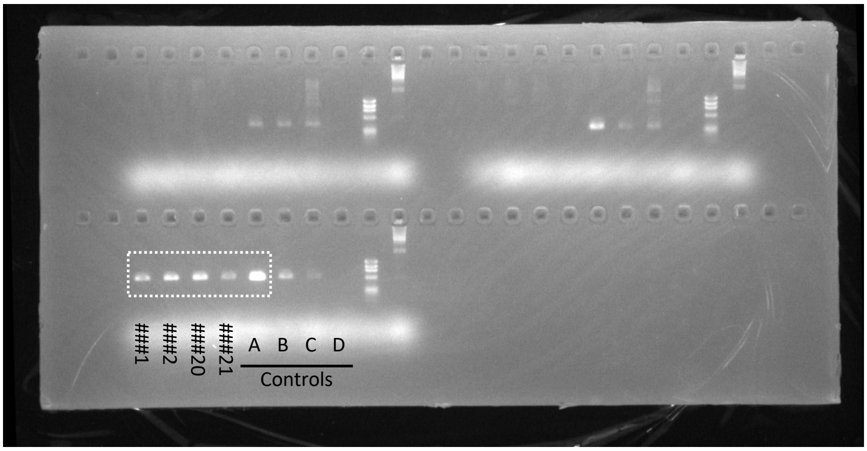

A: #99(PG/+)
B: PGK12.1-WT
C: PGK12.1-TK(+) #1
D: XGFP-TV

Fat60

Figure S6. Uncropped electrophoresis gels of Fig. 2C.

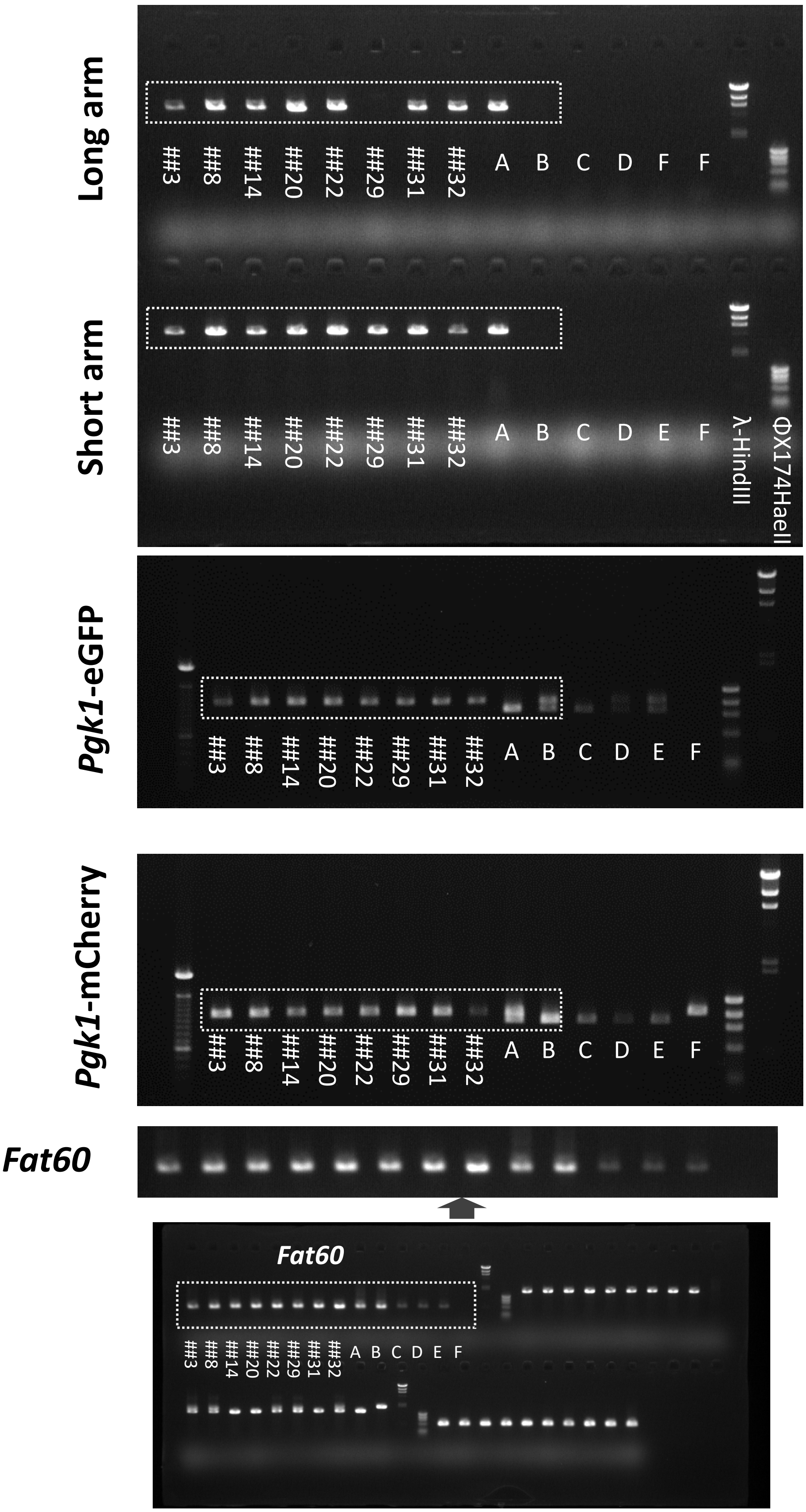

A: #874 (PR/+)
B: #99(PG/+)
C: PGK12.1-WT
D: PGK12.1-Green ###21
E: PGK12.1-TK(+) #1
F: XRED-TV

Figure S7. Uncropped electrophoresis gels of Fig. 2D.

Supplemental table S1

A

Primer	Sequence
F1	5'- ATCAGACTCTGGTCTTTGGCTTCTTCACGT -3'
R1	5'- GCTTTCCAGTCGGGAAACCTGTCG -3'
F2	5'- GCCTTCTATCGCCTTCTTGACGAGTTCTTC -3'
R2	5'- CCTTGCACATGAATCCAATGCACTGAATAC -3'
R3	5'- CGTGGAACAGTACGAACGCG -3'
F3	5'- GCAACCTCCCCTTCTACGAGC -3'
F4	5'- GAACGAGATCAGCAGCCTCTGTTCCACATA -3'
R4	5'- GCCCTGAGCAAAGACCCCAACGAGA -3'
R5	5'- AGACAGGTCTCACTTGGTAGCCTTGGTCAG -3'
F5	5'- CGCCTCTACAGGAGGGTCTTGACC -3'
R6	5'- GAGCCATTTGGCAAACACTGGGC -3'
F6	5'- CACGAGTGGAAAGCAACTCA -3'
R7	5'- AGATGGTGGTCTGGCTGAAC -3'
F7	5'- CACCCACCCATGCTAGTCTT -3'
R8	5'- ACCCTCAAACTCCTGGTCCT -3'
F8	5'- GGCCAGTCCAGAATACCAGA -3'
R9	5'- GAACTCGCTTCCAGAACCTG -3'
F9	5'- ACAATCCCCTGAGACACAGC -3'
R10	5'- GCGACGTTTTCTTCGTCTTC -3'
F10	5'- AACTTTGGCATTGTGGAAGG -3'
R11	5'- ACACATTGGGGGTAGGAACA -3'
F11	5'- GCCTCAAGAAGAAGGATTGCCTGGA -3'
R12	5'- GCCTCAAGAAGAAGGATTGCCTGGA -3'

B

Primer set	Purpose
F1/R1	eGFP genotyping
F2/R2	eGFP genotyping
F1/R3	mCherry genotyping
F3/R2	mCherry genotyping
F4/R4/R5	eGFP genotyping
F4/R3/R5	mCherry genotyping
F5/R6	*Ftx* amplification
F6/R7	*Oct3/4* RT-qPCR
F7/R8	*Nanog* RT-qPCR
F8/R9	*Rex1* RT-qPCR
F9/R10	*FGF5* RT-qPCR
F10/R11	*Gapdh* RT-qPCR
F11/R12	*Xist* SNP locus amplification

Supplemental table S2

Oligo	Sequence
Top	5'- caccgCTTCTGATGGAATTAGAACT -3'
Bottom	5'- aaacAGTTCTAATTCCATCAGAAGc -3'

* oligos used for gRNA cloning.
